# Coronary atherosclerosis in athletes: emerging concepts and preventive strategies

**DOI:** 10.1093/eurheartj/ehae927

**Published:** 2025-01-10

**Authors:** Guido Claessen, Thijs M H Eijsvogels, Christine M Albert, Aaron L Baggish, Benjamin D Levine, Eloi Marijon, Erin D Michos, Andre La Gerche

**Affiliations:** Faculty of Medicine and Life Sciences, Biomedical Research Institute, LCRC, UHasselt, Hasselt, Belgium; Hartcentrum Hasselt, Jessa Ziekenhuis, Stadsomvaart 11, 3500 Hasselt, Belgium; Department of Cardiovascular Diseases, KU Leuven, Herestraat 49, 3000 Leuven, Belgium; Department of Medical Biosciences, Exercise Physiology Research Group, Radboud University Medical Centre, Nijmegen, The Netherlands; Department of Cardiology, Smidt Heart Institute, Cedars-Sinai Medical Center, Los Angeles, CA, USA; Division of Cardiology, Lausanne University Hospital, Lausanne, Vaud, Switzerland; Division of Cardiology, Department of Internal Medicine, University of Texas Southwestern Medical Center, 5323 Harry Hines Blvd., Dallas, TX 75390, USA; Institute for Exercise and Environmental Medicine, Texas Health Presbyterian Hospital Dallas, 7232 Greenville Ave, Dallas, TX 75231, USA; Paris Cardiovascular Research Center, Université Paris Cité, Inserm U970, Paris, France; Division of Cardiology, European Georges Pompidou Hospital, Paris, France; Division of Cardiology, Department of Medicine, Johns Hopkins University School of Medicine, Baltimore, MD, USA; Heart, Exercise and Research Trials (HEART) Lab, St. Vincent’s Institute of Medical Research, Melbourne, Australia; Department of Cardiology, St. Vincent’s Hospital Melbourne, Fitzroy, Australia; HEART Lab, Victor Chang Cardiovascular Research Institute, Darlinghurst, NSW 2010, Australia

**Keywords:** Athletes, Coronary atherosclerosis, Ischaemic heart disease, Exercise, Sudden cardiac death, Coronary computed tomography angiography, Cardiovascular risk

## Abstract

There should be no assumption that an athlete is immune to coronary artery disease (CAD), even when traditional cardiovascular (CV) risk factors appear well-managed. Excelling in certain aspects of health does not equate to total CV protection. Recent data from cardiac imaging studies have raised the possibility that long-term, high-volume, high-intensity endurance exercise is associated with coronary atherosclerosis. Whilst the risk of CV events has not been shown to rise with athletic activity, the potential for CAD should not be overlooked as it is the leading cause of sudden cardiac death in athletes >35 years of age (i.e. ‘Masters athletes’). Evaluating both traditional and non-traditional risk factors for CAD is the most important part of pre-participation evaluation in Masters athletes. When managing athletes at risk of CAD it is important to adopt a shared decision-making approach regarding lifestyle adaptation and lipid-lowering treatments. In the great majority of athletes, after excluding the presence of symptoms and inducible ischaemia, this advice should include encouragement to continue exercising as available data indicate that higher levels of fitness are associated with a markedly attenuated incidence of coronary events regardless of the severity of coronary disease. Future research is needed to establish the relationship between clinically relevant CAD outcomes and coronary artery calcification in Masters Athletes, the role of sex, as well as exploration of the mechanisms underpinning these unexpected CV adaptations.

## Graphical Abstract

**Graphical Abstract:**
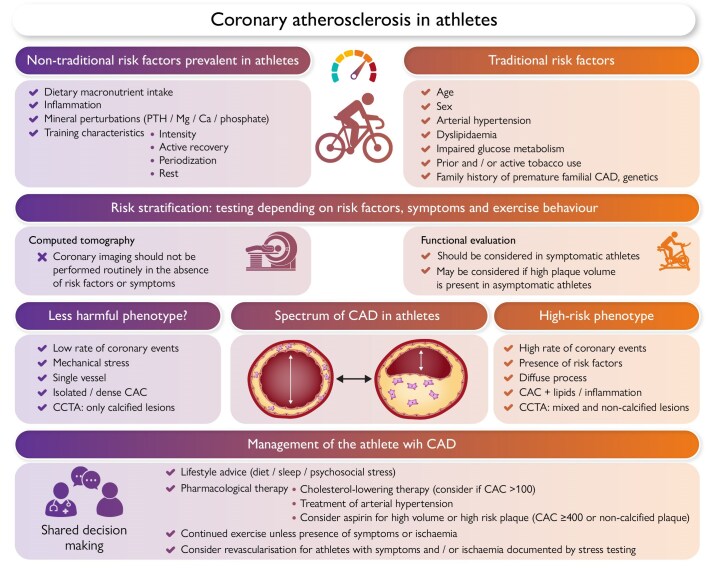
Evaluation and management of athletes at risk of coronary atherosclerosis. CAC, coronary artery calcification; CAD, coronary artery disease; CCTA, coronary computed tomography angiography; PTH, parathyroid hormone.

## Introduction—case illustration

The dose–response relationship between long-term high-intensity endurance sports and coronary heart disease has intrigued investigators for decades and remains a matter of debate. A sentinel paper examining autopsy data of marathon runners who died from traumatic causes proposed that the ability to run 42 km confers immunity from ischaemic heart disease.^1^ However, subsequent work has refuted this premise and there are recent observations which suggest that high levels of athletic training may promote coronary artery disease (CAD). The following case vignette highlights the importance of this review topic as it pertains to addressing the unconscious bias that could result in the under-appreciation of symptomatic CAD in an elite athlete. We propose that athleticism and CAD should not be seen as mutually exclusive and that high-level endurance exercise training may ultimately prove to be a coronary disease risk factor.

**Figure 1:**
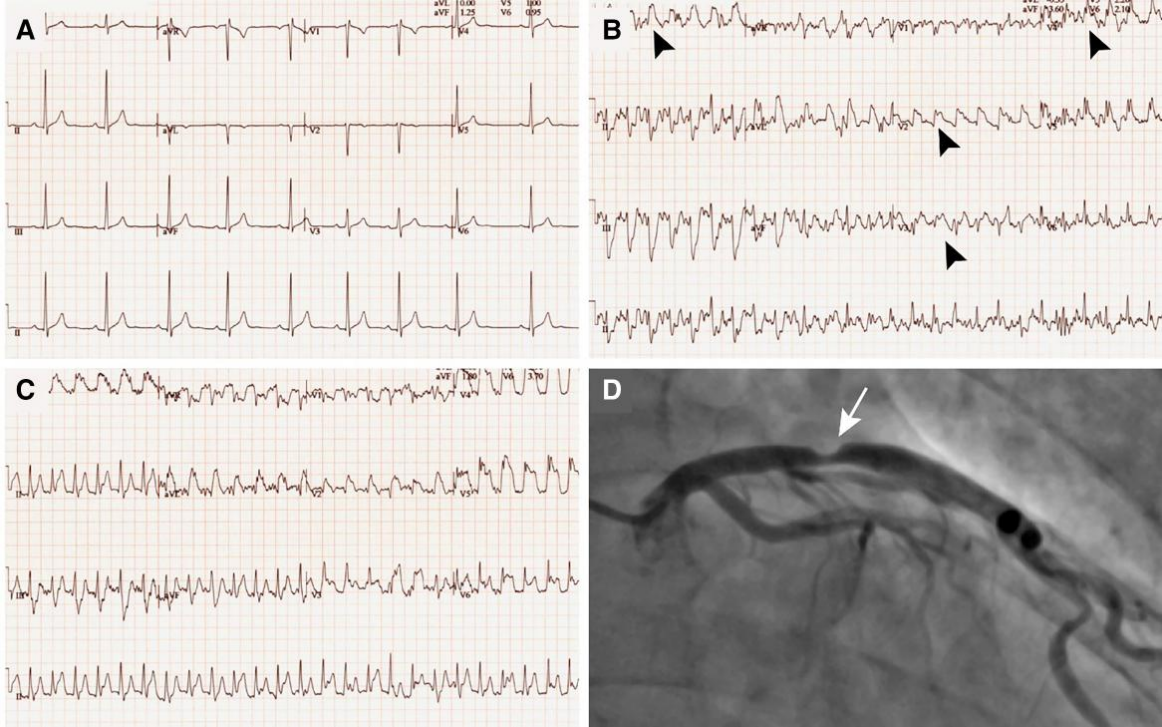
Case presentation of a 44-year-old previous Tour de France cyclist with chest pain and abnormal exercise stress test. Resting 12-lead electrocardiogram (*A*), electrocardiogram during exercise (*B*), and early recovery (*C*) showing manifest ST-segment elevation (black arrowheads) in leads V2–V6 and leads I and aVL. (*D*) Coronary angiogram with a significant proximal left anterior descending lesion (white arrow)

A 44-year-old previous Tour de France cyclist was assessed at the outpatient clinic because of left shoulder pain occurring whilst riding steeper hills whilst training a national team. Several days before his consultation, he had presented to the emergency department because of similar symptoms but had been dismissed based on his low cardiovascular (CV) risk profile and negative work-up, including a normal electrocardiogram (ECG) (*Figure 1A*) and troponin levels. There was no significant family history nor traditional CV risk factors. He was normotensive and his lipid profile was within the normal range. Resting echocardiography revealed typical features of the athlete’s heart phenotype, but no evidence of an underlying cardiomyopathy nor the presence of regional wall motion abnormalities. Exercise ECG revealed inducible ST-segment elevation in leads V2 to V6 and leads I and aVL during peak exercise (*Figure 1B*) and recovery (*Figure 1C*), suggestive of substantial myocardial ischaemia in the proximal left anterior descending (LAD) artery . A coronary angiogram (*Figure 1D*) was performed and confirmed a significant proximal LAD lesion (>70% stenosis) for which percutaneous coronary intervention was performed.

The finding of clinically significant CAD in an ostensibly healthy athlete with no traditional risk factors may be viewed as surprising.^2,3^ However, recent cardiac imaging studies raise the possibility that long-term, high-volume, high-intensity endurance exercise may actually accelerate rather than reduce coronary atherosclerosis.^4–7^ This controversial topic triggers many questions that are vital in the care of highly active individuals. For example, what is the causal relationship between long-term endurance exercise training and the risk of coronary atherosclerosis? To what degree does coronary artery calcification (CAC) in Masters athletes truly reflect the presence of atherosclerosis as defined by the presence of plaques containing lipids and inflammatory cells? If exercise is the underlying culprit, how do we then explain that some athletic individuals develop significant coronary plaques, whereas others have no plaques despite a similarly low CV risk profile? Are women less affected by coronary plaques in relation to exercise? Can differences in plaque development be attributed to differences in training load and sports specificities or do other modifying factors play a role? If so, how do we advise athletic people such as the one highlighted in our case presentation regarding future sports prescription? These are very important questions because exercise is a powerful medicine that prevents many chronic diseases,^8^ including CAD, and exercise restriction may cause more harm than benefit. A future scientific priority is to unravel the pathophysiological mechanisms underpinning coronary alterations in athletes and their clinical implications. This insight will be necessary to delineate effective risk stratification and preventive strategies. We will discuss the role of current risk-stratification tools in clinical practice and whether findings such as elevated coronary artery calcification (CAC) should be interpreted in the same way in athletes as in the general population.

## Coronary atherosclerosis in athletes—what is the evidence?

**Figure 2:**
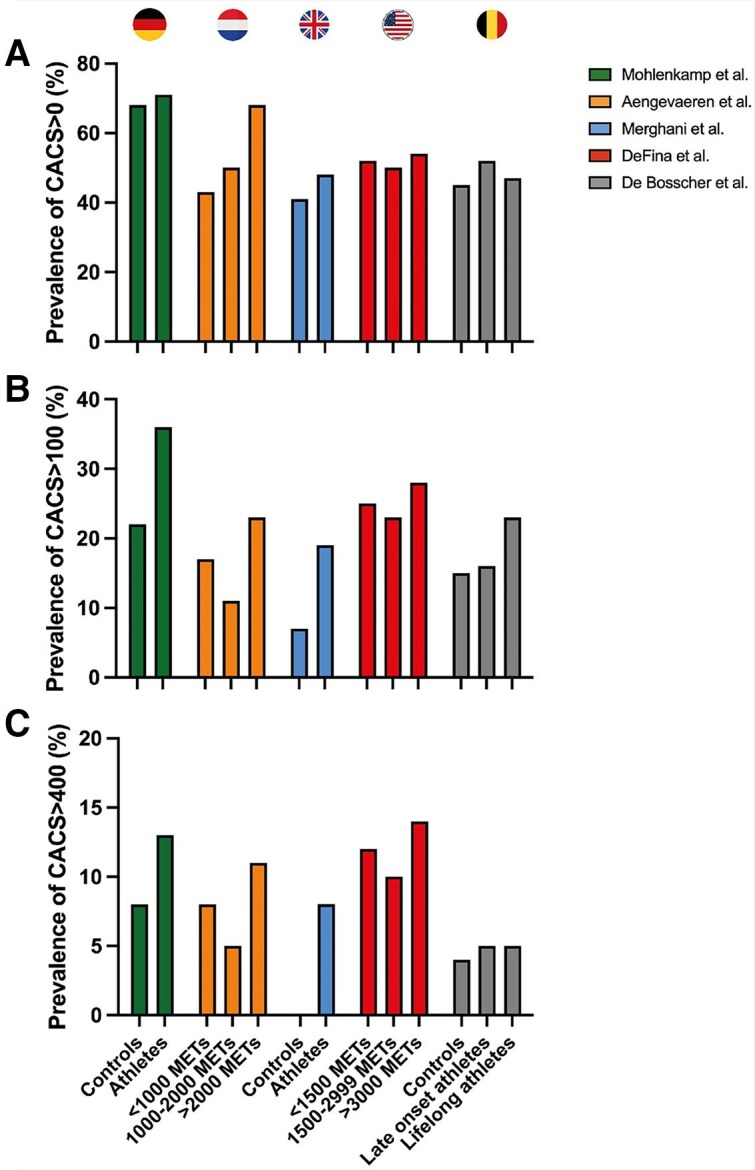
Prevalence of coronary artery calcification scores in studies comparing male athletes with controls.^4–7,9^ Prevalence of coronary artery calcification scores >0 within athletic and control subjects (*A*). (*B*) Prevalence of coronary artery calcification scores >100. (*C*) Prevalence of coronary artery calcification scores >400

Regular physical activity is a well-established way of preventing CAD,^3^ but several international cohort studies have reported an increased prevalence of coronary calcification and plaques amongst highly trained male athletes compared with less active people.^4–6,9^ These studies have offered significant insights into the relationship between coronary atherosclerosis and exercise training, but the observational nature of these studies warrants careful interpretation of the findings. Key strengths and limitations of the different studies are highlighted in *Table 1*. The landmark paper by Möhlenkamp *et al.*^4^ reported higher CAC scores in marathon runners compared with risk factor-matched controls. The prevalence of a CAC score ≥100 was 36% in runners compared with 22% in age- and risk factor-matched controls (*P* = .01; *Figure 2*). In alignment with the data by Möhlenkamp *et al.*, the Cooper Center Longitudinal Study (CCLS) also found increased risk for CAC > 100 in individuals with higher levels of habitual physical activity.^9^

**Table 1:** Overview of studies on the relationship between coronary atherosclerosis and exercise training

Study	Subgroups	Sample size	Age	Exercise characteristics	Smoking (%)	CAD family history?	VO2max, mL/kg/min (%)	Key strengths/weaknesses^b^
Mohlenkamp^4^	Controls	108	57.1	1389 METmin/wk	Current smoking 4.6% former smoking 51.9%	NA	NA	Strength: first alert to prevalent CAD in athletes weakness: high prevalence of CV risk factors in athletes
	Athletes	216	57.2	4686 METmin/wk	Current smoking 4.6% former smoking 51.9%	NA	NA	
Aengevaeren^6^	<1000 METs	88	54.4	669 METmin/wk	Current smoking 8% former smoking 36%	33%	NA	Strength: dose–response of exercise and CAC well-defined weakness: absence of a control group
	1000–2000 METs	121	54.8	1443 METmin/wk	Current smoking 4% former smoking 60%	29%	NA	
	>2000 METs	75	55.9	2724 METmin/wk	Current smoking 3% former smoking 44%	33%	NA	
Merghani^5^	Controls	54	52.5	1.9 h/week	Never smokers	24.1%	30.9 (96%)	Strength: low prevalence of CV risk factors and well-matched controls weakness: modest sample size
	Athletes	106	55.1	7.5 h/week	Never smokers	15.1%	44.4(133%)	
DeFina^9^	<1500 METs	16 447	49.5	474 METmin/wk	Current 15.1% former smoking NA	NA	38.6^a^	Strength: large sample size and inclusion of women weakness: no angiography
	1500–2999 METs	3750	49.2	2078 METmin/wk	Current 10.5% former smoking NA	NA	43.3^a^	
	>3000 METs	1561	50.0	4618 METmin/wk	Current 12.0% former smoking NA	NA	45.1^a^	
De Bosscher^7^	Controls	191	55	888 METmin/wk	Never smokers	6.3%	37 (122%)	Strength: comparison of lifelong athleticism vs. onset at middle age, recruitment strategy weakness: very active controls
	Late-onset athletes	191	55	4884 METmin/wk	Never smokers	6.8%	46 (155%)	
	Lifelong athletes	176	56	5070 METmin/wk	Never smokers	6.3%	48 (159%)	

^a^Estimated based on maximal time and grade on an exercise treadmill; Additional data providing further characterization of CAC scores in the CCLS data provided by Laura deFina.

^b^Limitation to all studies is the absence or small cohort representation of women and the lack of documentation of dietary confounders.

**Figure 3:**
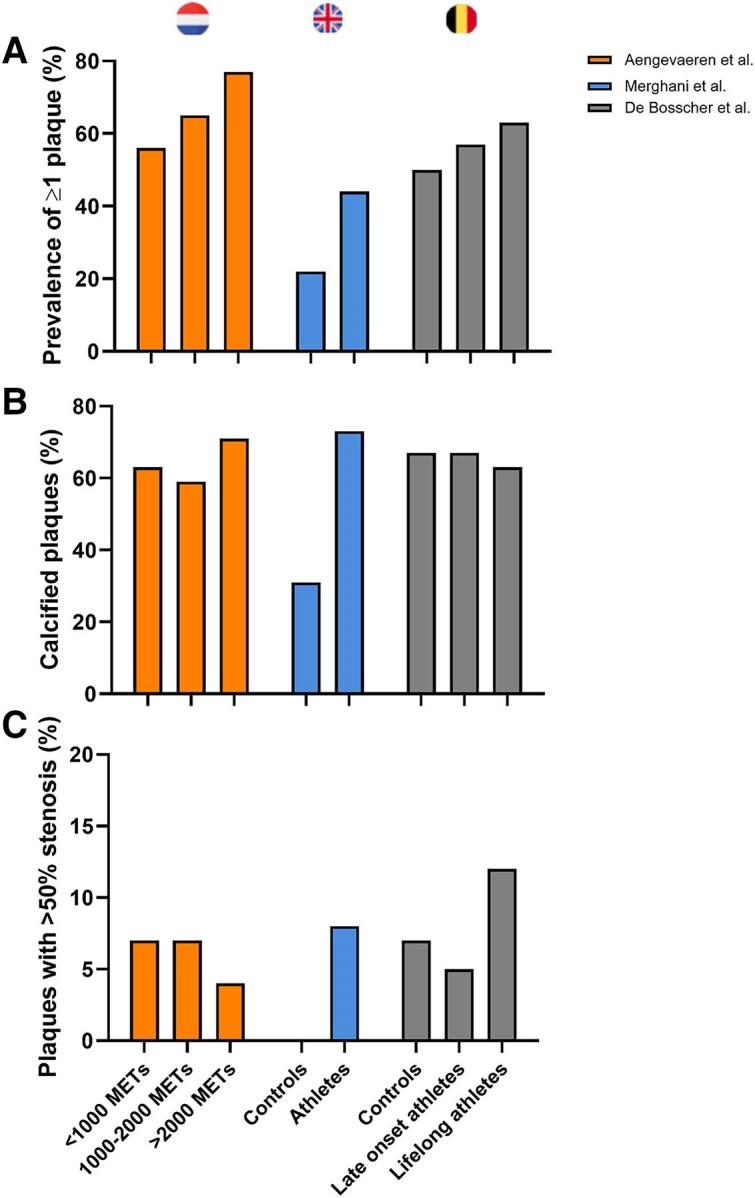
Prevalence of coronary artery plaques in studies comparing male athletes with controls.^5–7^ Percentage of athletic and non-athletic individuals with ≥1 coronary artery plaque (*A*). (*B*) Percentage of calcified plaques in individuals with coronary plaques. (*C*) Percentage of coronary plaques with >50% stenosis amongst individuals with coronary plaques

In addition to CAC scoring, three studies used coronary computed tomography angiography (CCTA) to investigate the relationship between exercise training and both plaque composition and the degree of luminal stenosis (*Figure 3*).^5,7,10^ Amongst 284 male recreational athletes from the Measuring Athlete’s Risk of Cardiovascular Events (MARC-1) study (aged ≥45 years), higher CAC scores and a higher prevalence of coronary plaques were reported in athletes with the highest lifelong exercise volume [>2000 metabolic equivalent of task (MET) min/week] and those with the highest dose of very vigorous exercise (≥9 MET).^6^ In the follow-up MARC-2 study, the progression of coronary atherosclerosis and its relationship with exercise characteristics over time was evaluated. Exercise intensity but not volume was associated with the progression of CAC and (mainly calcified) plaques during 6-year follow-up. Very vigorous exercise intensity (>9 METs) was associated with greater CAC and calcified plaque progression, whereas vigorous-intensity exercise (<9 METs) was associated with less CAC progression.^11^ A recent cross-sectional analysis from the CCLS showed that a higher weekly duration of activity was associated with more CAC, but a higher average intensity of activity was associated with less CAC in this population.^12^ Thus, more research is needed before final conclusions can be made regarding the impact of differences in training characteristics on coronary atherosclerosis.

The second study compared 106 male Masters athletes (aged 55 ± 9 years) without CV risk factors compared with 54 non-athletic controls.^5^ This study also included a comparison between 46 female Masters athletes and 38 non-athletes. Although the modest sample size was a limitation of this study, a particular strength was the complete absence of CV risk factors, including a family history of premature CAD, diabetes mellitus, hypertension, hypercholesterolemia, and active or prior tobacco smoking. Overall, there were no significant differences between athletes and controls with respect to the proportion with CAC >0 (48% vs. 41%, *P* = .33). However, despite an equally low CV risk profile, male athletes had a higher prevalence of coronary atherosclerotic plaques compared with controls (44% vs. 22%, *P* = .009) with plaques composed predominantly of calcium.^5^ The number of years of training was the only independent variable associated with increased risk of CAC >70th percentile for age or luminal stenosis ≥50% in male athletes.

Direct visualization of the coronary arteries using CCTA as performed in the Dutch and UK cohorts, rather than just measuring CAC, is clinically relevant as non-calcified and mixed plaques carry a greater risk of major acute adverse cardiac events than calcified plaques.^13^ In both studies, athletes had more calcified, less mixed, and a similar proportion of non-calcified plaques compared with non-athletes, thereby suggesting that exercise training may be associated with the development of a less harmful plaque composition. Based on these findings, it was hypothesized that accelerated calcification may represent a means of plaque stabilization which would fit with the observation that higher cardiorespiratory fitness is associated with a lower risk of CV events.

An important confounder in the majority of studies on coronary atherosclerosis in Masters athletes is the presence of CV risk factors, such as smoking or arterial hypertension (*Table 1*).^4,6,9^ Nevertheless, the inclusion of athletes with risk factors represents the real-world situation as data from the Boston MASTER (Masters Athletes Survey To Evaluate Risk) Initiative revealed that 64% of master athletes had at least one established CV risk factor with a family history of premature atherosclerotic disease and prior tobacco exposure identified as the most common issue.^14^ On the other hand, the high prevalence of risk factors makes it difficult to establish a definite causal link between endurance exercise itself and coronary atherosclerosis. The presence of risk factors may have induced compensatory lifestyle changes, such as initiation of exercise training in middle age, with a subsequent improvement of the CV risk profile. For this purpose, the MARC-2 investigators quantified lifelong exercise patterns and demonstrated that athletes in the most active group had been engaged in more exercise throughout their lifetime.^6^

To account for differences in starting age of exercise training, the Master@Heart study used CCTA to compare three groups: (i) 191 late-onset endurance athletes, who commenced training after the age of 30; (ii) 191 endurance athletes who had been exercising since a younger age; and (iii) 176 non-athletic controls.^15^ To address a potential for selection bias, volunteers were asked to register interest on an online platform from which study participants were randomly drawn. Similar to the UK cohort study, all Master@Heart participants had a low CV risk profile.^5,7^ Once again the seeming paradox between endurance exercise exposure and coronary atherosclerosis could be demonstrated; lifelong endurance athletes had more coronary plaques than fit and healthy individuals despite a similarly low CV risk profile (*Figure 3*). However, contrary to previous studies, the excess in coronary disease could not just be attributed to a greater burden of calcified lesions as a similar distribution of plaque composition was observed amongst the three groups.^7^

**Figure 4:**
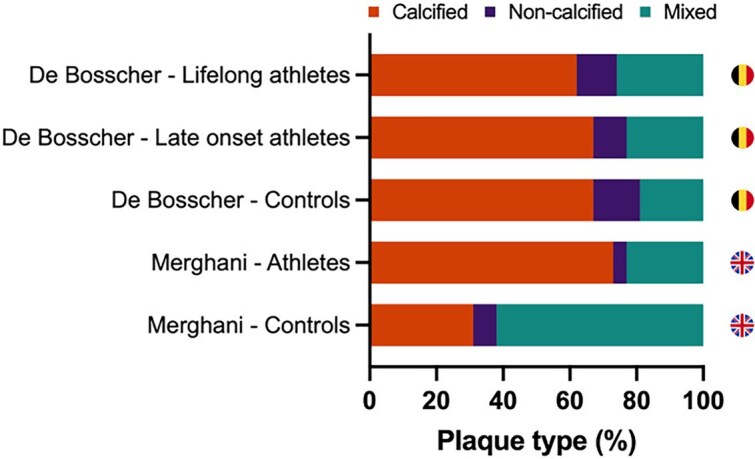
Distribution of plaque type in male athletes and non-athletic controls of the Master@Heart study^7^ and the paper by Merghani *et al*.^5^

When interpreting apparent discrepancies in plaque composition across athletic cohorts, it is important to consider the inclusion criteria.^7^ Controls in the Master@Heart study were allowed to perform up to 3 h of exercise per week and 77% percent performed leisure-time physical activity. As such, the Master@Heart control group constitutes a group of healthy individuals, which is further reinforced by their very low age-specific CAC percentile scores, benign plaque composition (67% calcified plaque), and relatively high fitness level (*Table 1*).^7,16^ Together with the absence of CV risk factors, these characteristics may explain why the plaque composition of the Master@Heart controls was comparable to the Masters athletes of the study by Merghani *et al*.^5^ (*Figure 4*) and the most active group of the MARC study,^6^ suggesting that all participants of the Master@Heart study had predominantly favourable plaque morphology. In contrast, the male control subjects from the UK study and the least active group of the MARC study were less fit and had a proportionately greater amount of mixed and non-calcified plaques.^5,6^

**Figure 5:**
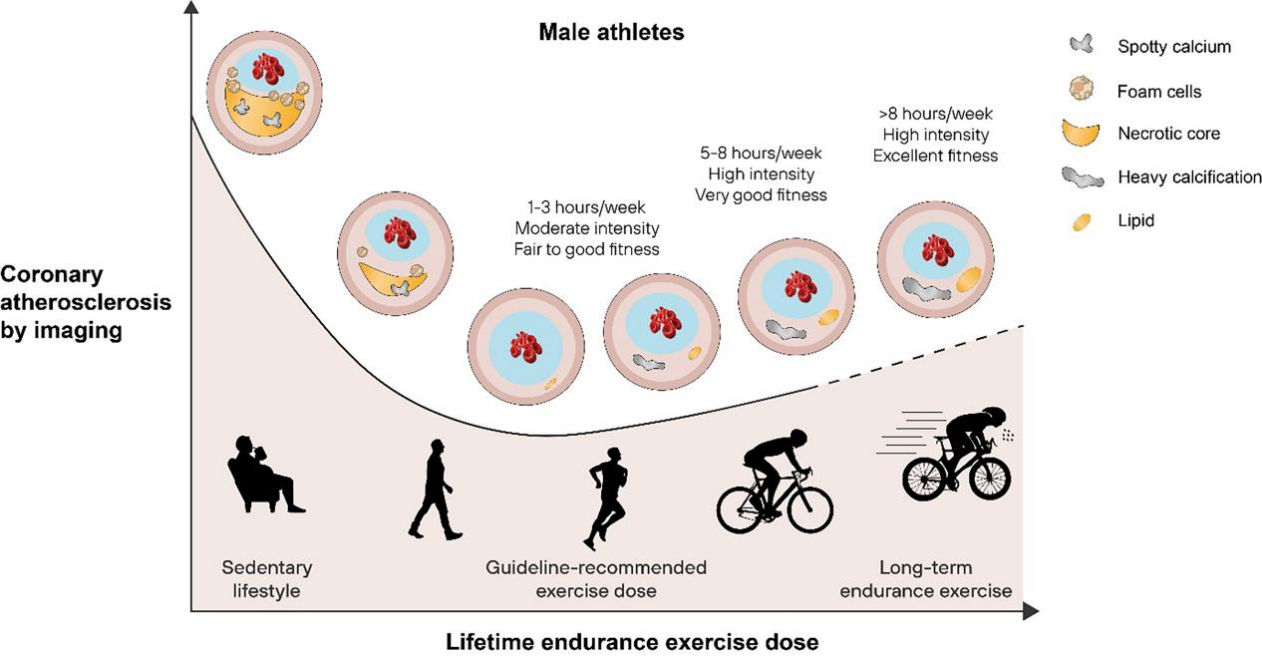
The relationship between lifetime endurance exercise dose and coronary atherosclerosis by non-invasive coronary imaging in male athletes^4–7^

Based on these studies, it appears that the dose–response relationship between endurance exercise and the prevalence of coronary atherosclerosis by non-invasive imaging may be reverse J-shaped rather than a descending curvilinear function (*Figure 5*). However, rather than just looking at the prevalence of coronary plaque and the degree of stenosis, it is also important to consider the magnitude of total coronary plaque burden because the latter may be a stronger predictor for CV events and death.^17^ Patients with a comparable calcified atherosclerosis burden generally carry a similar risk for ischaemic events regardless of whether they have nonobstructive or obstructive CAD.^17^ In both the UK and the Master@Heart cohorts the prevalence of individuals with CAC ≥400, indicating a large coronary plaque burden, was lower compared with other population studies in asymptomatic individuals with CV risk factors.^18^ Thus, whilst long-term intensive exercise training may cause an increase in coronary calcification and plaque burden, the magnitude of this effect may be relatively modest when compared with the effect of CV risk factors in the general population. A comparison of the relative risk associated with standard coronary risk factors vs. the effect of exercise has not yet been performed and may help provide context and balance.

In addition to total plaque burden, the appreciation of plaque risk in athlete studies was mainly confined to the differentiation into calcified, non-calcified, and mixed plaques. In contrast, limited data exist on the prevalence of imaging features that have been associated with plaque vulnerability, such as low attenuation, spotty calcification, positive remodelling, and the napkin sign. In the MARC study, low attenuation plaques were less prevalent in the most active group.^6^ Similarly, a lifelong athletic lifestyle was associated with a lower risk of vulnerable plaques, i.e. presence of ≥2 high-risk features, in the Master@Heart study.^7^ Future research is required to provide insights into the quantification and characterization of coronary plaque in the whole coronary tree,^19^ as well as the presence of pericoronary adipose tissue^20^ and the influence of coronary artery diameter.^21^

## Potential aetiologies and modifying factors

The mechanisms by which exercise influences the development and progression of coronary atherosclerosis are not yet fully elucidated. Observational data from athletic cohorts, along with findings from experimental studies, have proposed potential aetiological factors and modifiers; however, these hypotheses remain largely speculative and unvalidated.^22^ To date, most studies have examined endurance athletes.^4–7,11^ The comparatively few studies that have assessed coronary morphology in strength-trained athletes have reported mixed findings.^23,24^ Considering the distinct physiological effects of endurance vs. resistance exercise on the CV system and differences in the potential use and type of performance-enhancing drugs (e.g. erythropoiesis-stimulating agents vs. anabolic steroids) that may affect the development of coronary atherosclerosis,^25^ this section focuses exclusively on endurance exercise. Similarly, the influence of race is not considered as published studies predominantly included White athletes, despite the well-known impact of race and ethnicity on the risk of coronary atherosclerosis^26,27^ and its potential effect modification with habitual physical activity.^28^

Heart rate, blood pressure, circulating catecholamines, and coronary blood flow are directly related to exercise intensity.^29^ Accordingly, high-intensity exercise may lead to disruption of laminar coronary flow patterns,^30^ and increases in the magnitude of shear stress on the coronary walls. These factors have been associated with endothelial dysfunction, increased production of reactive oxygen species, formation of fatty streaks, and atherosclerotic plaque formation.^31–33^

Acute bouts of exercise also impact the immune system, evidenced by mobilization of leukocytes and an increase in circulating inflammatory mediators,^34^ such as tumour necrosis factor alpha (TNFα), interleukins (IL) IL-1β, IL-6, IL-8, and IL-10.^35–37^ The magnitude of this response depends on exercise intensity, with greater responses at high vs. moderate intensity exercise.^38^ Given the significant role of inflammation in the pathogenesis of coronary atherosclerosis,^39^ it is hypothesized that high-intensity, high-volume exercise may generate a proinflammatory environment that could potentially accelerate the progression of coronary atherosclerosis. However, this hypothesis remains unproven, as no empirical evidence currently supports a causal link between immunogenicity, inflammation, and an elevated risk of disease progression in athletes.^40^

Exercise-induced increases in concentrations of mediators of calcium homeostasis may also be relevant. Parathyroid hormone (PTH) is a key regulator of calcium and phosphate homeostasis. Elevated PTH, calcium, and phosphate concentrations are predictive of arterial calcification^41,42^ and may link exercise exposure to coronary calcification. Exercise volume and intensity-dependent thresholds have been observed to induce changes in circulating PTH concentrations,^43^ with marked increases following high-intensity endurance exercise but not after shorter or less intense bout of exercise.^44^ The role of PTH and other mediators of calcium phosphate regulation in the development of coronary atherosclerosis amongst athletes represents an important line of inquiry for future research.^45^

Dietary habits impact the development and progression of CAD.^46^ Master athletes report a higher energy intake compared with the general population, with higher absolute fat and carbohydrate intake.^47,48^ Dietary sugars, in particular rapidly digested sugars and fructose-enriched juice have been associated with higher CAD risk.^49^ The impact of regular consumption of energy drinks and gels during exercise on CV health in athletes and its potential influence on the development of coronary atherosclerosis remains unestablished. More research into the dietary characteristics of athletes and the potential link with atherosclerosis is needed.

Genetics have a strong influence on coronary atherosclerosis. For example, a twin study showed a strong genetic heritability of CAC scores and calcified plaque volumes.^50^ In addition, the progression of coronary atherosclerosis was dependent on genetic factors independent of baseline risk factors and CAC score amongst participants of the Epidemiology of Coronary Artery Calcification (ECAC) study.^51^ To date, over 160 genome-wide loci have been associated with CAD risk.^52^ The high prevalence of a positive family history of CAD (49.3%) amongst young individuals (<50 years) with coronary atherosclerosis further reinforce the importance of genetic risks.^53^ Whilst there is no evidence indicating genetic differences amongst athletes concerning the development of atherosclerosis, exercise training may interact with genetic factors to influence the progression of coronary atherosclerosis. A UK Biobank study showed that cardiorespiratory fitness, but not physical activity volumes, could attenuate CAD risk amongst individuals with intermediate and high genetic risk scores.^54^ In fact, a U-shaped association was observed for physical activity and CAD risk. Future studies that aim to assess the interaction between genetic traits and environmental modifiers, including exercise training, are warranted.

Polygenic risk scores (PRSs) for CAD may provide a more nuanced understanding of the genetic contributions to the development of coronary atherosclerosis in endurance athletes, surpassing the insights offered by self-reported family history.^55^ Recent research has robustly shown that polygenic risk is significantly correlated with long-term plaque progression and the presence of high-risk plaques in individuals suspected of CAD.^56^ However, there is a lack of data on the application of CAD PRS in athletes who have coronary atherosclerosis identified through imaging. Assessing lipoprotein(a) [Lp(a)] levels could be valuable in this context, as Lp(a) is considered an inherited risk factor for atherosclerotic cardiovascular disease (ASCVD), especially in younger individuals. A prospective study involving serial CCTA with a 10-year follow-up period demonstrated that patients with elevated Lp(a) levels exhibited a greater coronary plaque burden at baseline and experienced more rapid progression of coronary plaque compared with those with lower Lp(a) levels.^57^ However, the relationship between coronary atherosclerosis and Lp(a) levels in athletic populations remains to be investigated.

## The role of sex and cardiovascular risk in the endurance athlete

In the general population, men typically develop CV disease at a younger age and demonstrate a higher risk of CAD compared with women. Mortality rates are also generally higher in middle-aged men than in women of the same age, a trend that may persist throughout life.^58^ Amongst athletes, most data linking increased coronary plaque burden are derived from studies of middle-aged, White males, with limited evidence available for women due to smaller cohort sizes. In a study of 26 female marathon runners who had run at least one marathon per year for 10–25 years (average of 47 marathons per person), female endurance athletes exhibited a lower prevalence of coronary artery plaque compared with sedentary women who were clinically referred for CCTA. When coronary plaque was present in these athletes, it was related to older age.^59^ In a similarly small study involving 46 female Masters endurance athletes and 38 relatively sedentary female controls, no significant differences were observed between the two groups in terms of CAC prevalence, CAC score, or the number of coronary plaques.^5^ In a larger study of 196 female athletes compared with 59 relatively sedentary control females (mean age 54 in both groups) who underwent CCTA, there was again no increase in the prevalence of CAC, or differences in coronary plaque burden, or coronary plaque type.^60^ It should be noted that most of the female athletes in this study had a CAC score of 0 (prevalence of CAC >0 was 21% in athletes and 32% in controls, *P* = .07). Finally, data from the CCLS in 5341 women with direct measures of cardiorespiratory fitness and CAC scanning did not reveal any association between fitness and the presence of CAC when adjusted for traditional CV risk factors.^61^ The women with the lowest fitness did have a higher prevalence of CAC, but they also had more traditional risk factors than those women with the highest fitness.

The present data suggest that high-volume high-intensity exercise in females does not result in accelerated atherosclerotic changes, as previously reported in males. Instead, when coronary atherosclerosis is observed in Masters female athletes, it appears to be predominantly influenced by age and conventional CV risk factors. However, caution is warranted due to the smaller sample size relative to studies in male athletes. Additionally, women of similar age often demonstrate a lower prevalence of traditional CV risk factors and a reduced estimated 10-year risk for ASCVD,^58,62^ which may have contributed to their lower incidence of atherosclerosis. Consequently, whilst the findings are reassuring regarding coronary disease in female athletes, they underscore the necessity for larger, focused studies, particularly involving older female athlete cohorts.

Several mechanisms have been proposed to explain the observed ‘female advantage,’ suggesting a lower risk of accelerated coronary atherosclerosis in response to endurance training. However, these remain largely speculative, as the underlying mechanisms responsible for sex differences in coronary atherosclerosis amongst athletes have not yet been fully elucidated. Differences in sex hormone levels such as the lower levels of circulating androgens and higher oestradiol levels in females may be protective. Androgens, which are higher amongst males, may accelerate atherosclerosis or response to injury.^63^ In contrast, oestradiol, which is higher in females before menopause, has a vasodilatory effect on the coronary arteries^64^ and may protect against the development of coronary atherosclerosis.^65^ Furthermore, there are fundamental differences in body composition and anatomy between male and female athletes which translate to greater maximal oxygen consumption and cardiac outputs in male athletes.^66^ Finally, there may also be differences in athletic training between the sexes with a tendency for lower intensity of training for female’s endurance and strength training compared with male counterparts.^67^ It is possible that these factors would translate to the lower burden of haemodynamic shear stresses in women.

## Clinical manifestations of coronary atherosclerosis in athletes

**Figure 6:**
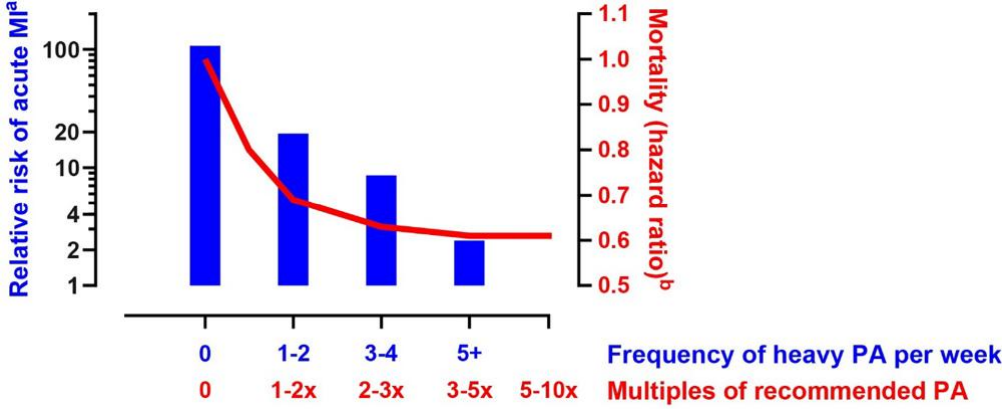
Relationship between habitual heavy physical activity (PA) and acute risk of acute myocardial infarction during acute vigorous exercise bouts (left *y*-axis). The right *y*-axis depicts the mortality hazard ratio estimates of exercise levels compared with the recommended minimum leisure-time physical activity of 7.5 metabolic equivalent hours per week. Adapted from data by Mittleman *et al.*^68^ and Arem *et al*.^69^

Physical activity and exercise training are associated with a significantly lower risk of CV disease and events, including a lower incidence of sudden cardiac death (SCD). In contrast to this long-term benefit, an acute bout of vigorous physical activity transiently increases the risk of dying suddenly (*Figure 6*), and this risk is particularly significant in untrained individuals with sub-clinical heart disease, including CAD.^70–72^ Coronary events in the context of atherosclerotic CAD remain the most common cause (up to 80%) of sports-related SCD in the community.^73,74^ The typical profile of sports-related SCD afflicts middle-aged individuals, with a striking male predominance (>90–95% of cases), even after consideration of differences in participation rate according to sex, notably higher than observed in non-sports-related SCD.^73,75,76^ Further study is required to explain this sex disparity and to determine how an excess of coronary disease in athletes exposed to long-term high-intensity, high volumes sports practice impacts on the favourable CV outcomes and longevity that have been ascribed to habitual exercise training in the general population.^2,11,77,78^

**Figure 7:**
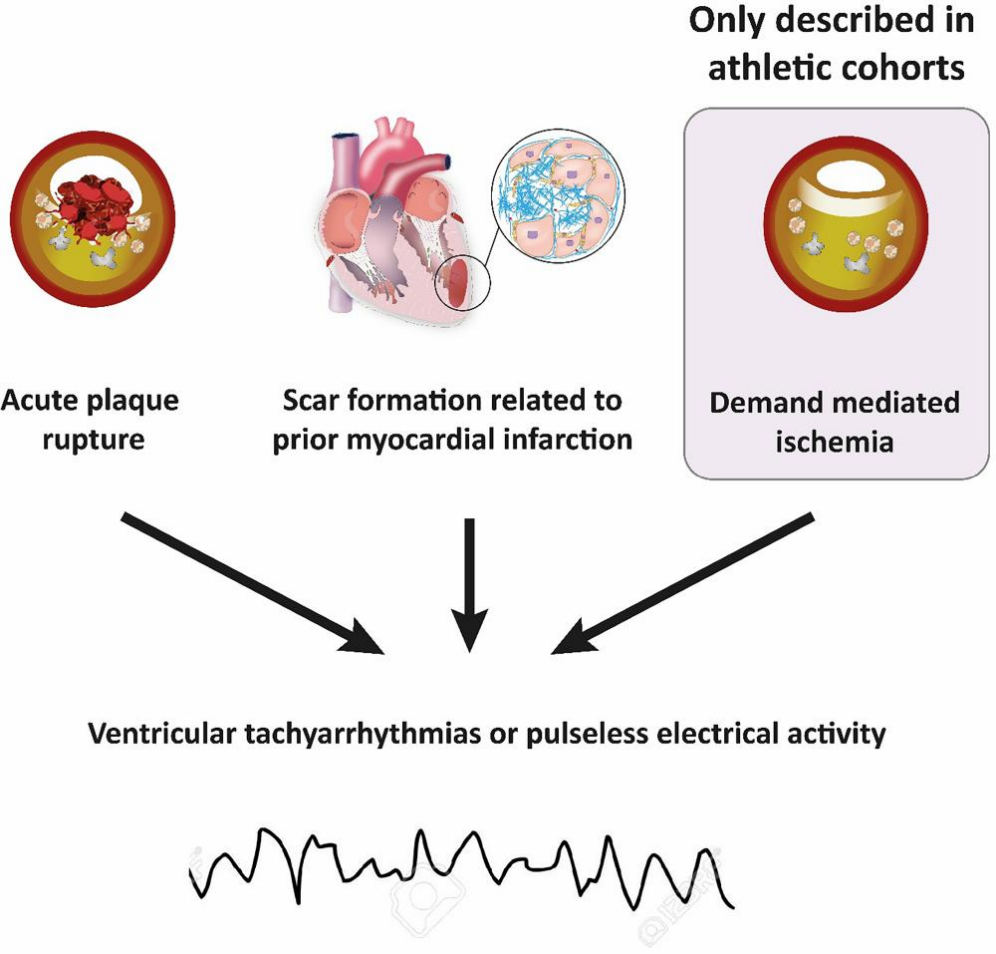
Mechanisms of sports-related sudden cardiac death due to coronary artery disease.^79–81^ Demand ischaemia is a recognized mechanism for ventricular arrhythmias and/or sudden cardiac death in athletes, driven by the myocardium’s work/metabolic requirements during intense exercise

CAD-related SCD presents in three ways (*Figure 7*): (i) acute myocardial infarction due to plaque rupture, (ii) demand-mediated ischaemia triggering polymorphic ventricular tachycardia or ventricular fibrillation, and (iii) re-entrant tachy-arrhythmias related to scar formation secondary to previous infarction or chronic ischaemia.^79^ The latter most commonly relates to the degeneration of scar-related monomorphic ventricular tachycardia into ventricular fibrillation or pulseless electrical activity and usually can occur years after previous myocardial infarction.^82^ In the setting of acute coronary syndromes, lethal ventricular arrhythmias have been reported to occur in more than 5–10% of cases occurring at rest,^83^ but this prevalence may be even higher during sports. Hence, acute bouts of vigorous physical exertion have been shown to transiently increase the risk of acute myocardial infarction and SCD,^68^ and men with exercise-induced SCD are more likely to have underlying plaque rupture on autopsy than those who had SCD at rest.^80^ Isolated stable plaques and chronic lesions are found in ∼40–50% of all CAD-related SCD.^81^ In these cases, acute demand-mediated ischaemia triggered by vigorous physical exercise is a plausible pathophysiological mechanism, and exercise training may actually decrease this risk.^80,81,84^

## Risk identification

The delineation of atherosclerotic CAD risk represents a fundamental goal in the care of the Masters athlete. As CAD is the most common cause of SCD during exercise amongst athletes above the age of 35,^73,74^ risk stratification should be performed for all Masters athletes. Risk stratification begins with an assessment of the presence, absence, and magnitude of traditional CAD risk factors. Age, sex, hypertension, dyslipidemia, impaired glucose metabolism, prior, and/or active tobacco use, and family history or premature familial CAD, as proposed and extensively validated in the 1998 Framingham risk score,^85^ represent the fundamental determinants of CAD risk. These factors can be easily assessed during routine clinical encounters thereby setting the stage for lifestyle intervention, pharmacotherapeutic intervention, and subsequent testing.

Numerous multivariable scores for the prediction of CAD risk that extend beyond the historical Framingham approach have been developed.^86^ Several of these scores have been integrated into clinical recommendations for the care of Masters athletes.^87,88^ Although a detailed comparison of risk scores is beyond the scope of this document, several general considerations for the practicing clinician are provided. Ideally, the choice of a specific CAD risk score should be individualized based on patient-specific characteristics including age, sex, and ethnicity. In addition, CAD risk scores that predict ‘hard’ outcomes including death and myocardial infarction are preferable to those that predict coronary revascularization. There are several specific considerations regarding the application of CAD risk scores amongst Masters athletes. First, most CAD risk scores define risks of adverse outcomes over relatively short periods of time (i.e. 10 years). Thus, these tools may incompletely capture lifelong risk in relatively young but ‘at risk’ athletes.^89^ Second, the majority of widely used CAD risk scores fail to capture some important factors that contribute to true overall risk including dietary macronutrient intake, psychosocial stress, and training approaches including rest, active recovery, and periodization. Finally, no available CAD risk score incorporates the impact of habitual physical activity on overall CAD risk. This represents a major limitation of quantitative CAD risk calculation in Masters athletes as the clear benefits of routine physical exercise have been extensively documented.^2,3^ Further work delineating the degree to which lifelong physical activity modulates overall risk in the presence of more commonly measured CAD risk factors represents a critical area of future research.

Diagnostic testing may provide incremental delineation of clinically relevant CAD risk. Computed tomography imaging to determine the presence and magnitude of CAC has emerged as a powerful and independently predictive risk stratification test in the general population.^90^ However, its use in Masters athletes remains controversial. Computed tomography coronary imaging (CAC scoring and/or CCTA) should not be performed routinely in the absence of risk factors or symptoms as data establishing associations between clinically relevant CAD outcomes and CAC have not been established for Masters athletes. It is possible that some Masters athletes will develop CAC simply from the repetitive mechanical stress of exercise on the proximal coronary vessels,^91^ rather than from the process of inflammatory, lipid-rich atherosclerosis. Coronary computed tomography angiography, ideally performed following abnormal functional evaluation for ischaemia, is preferable to CAC scoring in isolation, especially if the athlete has symptoms, or an abnormal response to exercise testing. The ability to differentiate presumably less harmful CAC from CAC associated with high-risk plaques provides valuable and actionable information about the importance of lifestyle modification, pharmacologic intervention, and consideration of revascularization. Ischaemia stress testing, ideally utilizing myocardial perfusion imaging or echocardiography in conjunction with exercise testing,^92^ should be performed in athletes with symptoms suspicious for myocardial ischaemia.

Care should be taken in the further evaluation of asymptomatic athletes with an elevated CAC (≥400). There is no evidence to support revascularization strategies in asymptomatic patients regardless of the degree of coronary atheroma and therefore it would be entirely reasonable to optimize preventive strategies and restrain from any further evaluations. However, in the authors’ experience, the finding of coronary atheroma in highly active athletes can promote a level of anxiety that can be difficult to fully address with counselling alone. Thus, functional evaluation of asymptomatic highly active athletes may be considered as a means of excluding significant ischaemia to fully reassure the athlete and enable them to continue to exercise and gain the preventive benefits associated with preserved fitness. The ‘risk’ of this strategy could be the finding of a large region of sub-clinical myocardial ischaemia which would create a difficult clinical conundrum with little evidence to inform decision making. In the authors’ experience, such situations are uncommon and may sensibly lead to invasive evaluation of the coronary arteries on the basis that a tight proximal lesion may be the substrate for ischaemia-driven coronary events at high levels of exertion, in accordance with current guidelines.^88,93^

## Management of coronary artery disease in the athlete

The possible association between lifelong endurance exercise training and the progression of CAD leads to questions regarding the best primary prevention strategies for the athlete with an elevated CAC score or findings of plaque on CCTA. There is little evidence to guide advice, but important inferences can be drawn from observational data linking exercise training to coronary events.

*Thank you for advising this 60-year-old competitive cyclist with a CAC score of 400* is a referral with which all sports cardiologists are becoming increasingly familiar. In addition to important pharmacological considerations, questions arise regarding the implications for ongoing exercise training and competition. It is important to recognize that regular exercise training is a potential trigger for acute coronary events, but also reduces the risk of exercise-related coronary events and has a net benefit in reducing CV and overall mortality.^94,95^ One side of the exercise paradox is more visible than the other. Cases of athletes experiencing a coronary event whilst exercising are well publicized and registered in the consciousness more than the greater numbers of deaths prevented by regular exercise.

**Figure 8:**
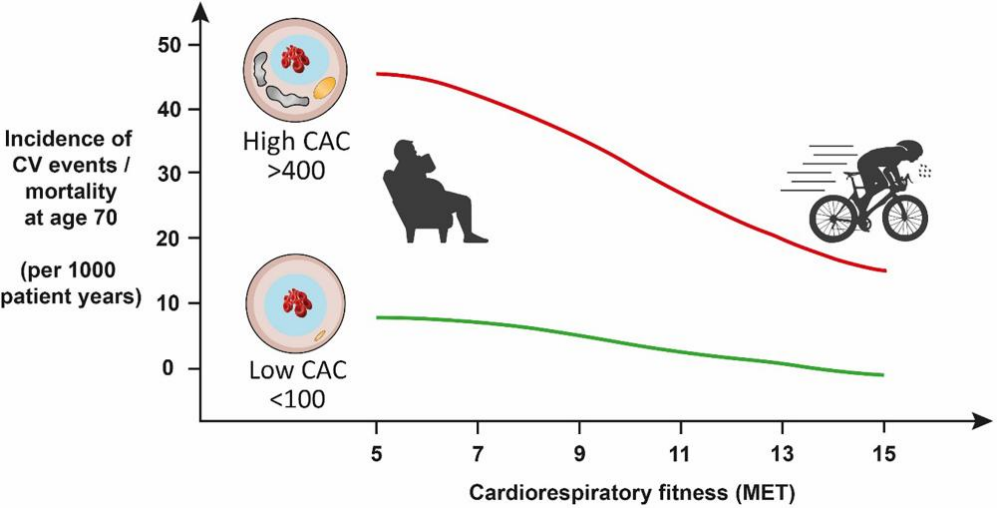
Cardiovascular event rates plotted with respect to cardiorespiratory fitness and coronary artery calcification score. Adapted from Radford *et al*.^96^

The protective effects of regular exercise are elegantly illustrated in a CCLS study assessing incident CV events over 25 years in 8425 men who underwent an assessment of CAC and cardiorespiratory fitness.^96^ The incidence of CV events increases with CAC score,^90,96^ but the pertinent finding relative to an athletic population is that this risk was attenuated with increasing baseline fitness (*Figure 8*). For example, in men with a CAC >400 at baseline, ∼50% of unfit men experienced a CV event over the following 25 years as compared with 20% of those with superior fitness.^96^ In a CCLS follow-up study amongst 21 758 men it also was noted that highly active men (>3000 METmin/wk) with CAC <100 had a significantly lower risk of CV and all-cause mortality compared with participants performing <1500 METmin/wk.^9^ However, this effect was attenuated in individuals with CAC ≥100 and it remains unclear whether this was due to insufficient statistical power or not. The CCLS data are limited by its observational nature and as this was not an interventional trial one cannot assume that encouraging individuals with an elevated CAC score to maintain exercise training will result in the observed risk reductions. However, until such a trial is conducted, these observational data provide a strong rationale for advising middle-aged exercise enthusiasts to maintain their fitness as part of the preventive treatment regime in those with an elevated CAC score.

Recent evidence showed that continued physical activity does not appear to accelerate the rate of coronary calcification.^97^ Amongst a population of 6661 men and 2110 women, the mean CAC score increased from 96 to 222 and from 20 to 48 Agatston units, respectively. The rate of change was not significantly influenced by the amount of exercise practiced in the 8-year follow-up period. These observations reinforce observations of the MARC-2 study as exercise intensity, rather than exercise volume, promoted CAC progression. Middle-aged athletes should, therefore, be encouraged to continue to exercise because (i) incident coronary events are less common amongst those who are fitter, and (ii) the progression of disease is only modestly impacted by exercise practise, if at all.

Preventive strategies in middle-aged athletes are not limited to advice regarding exercise. We recommend a multi-faceted preventive strategy that combines continued exercise with dietary advice, cholesterol-lowering therapy, and treatment of hypertension as dictated by the presence and magnitude of these well-established risk factors. This approach should be emphasized in athletes with evidence of coronary plaques that include any non-calcified tissue. At present, the initiation or intensification of pharmacotherapy based simply on CAC scoring amongst Masters athletes is not supported by any data. However, this approach may be reasonable given that individuals with high cardiorespiratory fitness and higher CAC levels have a higher risk of CV events than equally fit individuals with low or absent CAC.^96^ Risk scores that incorporate information from coronary imaging, such as that derived from the Multi-Ethnic Study of Atherosclerosis^98^ or the Astronaut Cardiovascular Health and Risk Modification (AstroCHARM) study,^99^ can be used to estimate incident CV events and the potential absolute benefit from instituting medical therapies. The relative benefits of therapies that have established efficacy in stabilizing coronary plaques and reducing clinical events will not be discussed at length in this review, but it is important to point out that many athletes will be more willing to consider a preventive regime that includes both lifestyle and pharmacological strategies if the clinician is able to include an informed discussion regarding the benefits of continued exercise. It is vital to engage in conversations about the possible side effects of medications that might affect athletic performance with the athlete. This aspect holds considerable weight, as it can greatly influence athletes’ willingness to start pharmacological treatments. For instance, it has been observed that individuals participating in high-intensity sports are more likely to experience statin-associated muscle symptoms compared with those engaged in recreational physical activities.^100^ In managing athletes, a systematic and graduated pharmacological strategy is essential. Innovative strategies, such as intermittent dosing and the utilization of non-statin or combined lipid-lowering therapies, may be required to effectively administer potent medications within the scope of lipid management.

The optimal management strategy for ischaemia in asymptomatic Masters athletes remains uncertain, necessitating a shared, informed decision-making process regarding revascularization. In the ISCHEMIA trial, major adverse ischaemic events were not reduced when patients with stable angina were treated with revascularization as opposed to optimal medical therapy.^101^ However, caution should be exercised before applying these findings to an athletic population. Although the optimal medical therapy in the ISCHEMIA trial included at least 150 min of moderate intensity physical activity per week, the exercise volume and intensity in this trial do not correspond to the levels typically achieved by athletes. There is evidence indicating that athletes may differ from the non-athletic population due to the potential risk associated with demand-induced ischaemia, driven by the myocardium’s work/metabolic requirements during intense exercise.^102^ Consequently, in the absence of studies specifically addressing revascularization in athletes with obstructive CAD, it may be prudent to consider revascularization in athletes presenting with symptoms and/or documented myocardial ischaemia during stress testing. Although definite evidence is lacking, this approach aligns with the current European Society of Cardiology recommendations on sports cardiology and exercise for patients with CV disease.^87,88^

## Conclusion

Decades of literature and extensive clinical experience have established that no level of fitness or habitual exercise confers immunity to CAD. Accumulated evidence supports a more educated, nuanced appreciation of the interaction between lifestyle factors and CAD. Some athletes appear to be at greater risk of sub-clinical coronary calcification and perhaps true atherosclerosis. This observation is supported by several studies with strikingly consistent findings but, importantly, the data are largely limited to cohorts of middle-aged, White men exposed to decades of intense, endurance exercise. Importantly, data establishing associations between clinically relevant CAD outcomes and CAC have not been established for Masters athletes. There is a clear need to expand the knowledge base to include more diverse populations. The other critical priority is to understand the relationship between the surrogate measures of CAC, coronary plaque, and the incidence of clinical CV events in athletes. In the meantime, the observational data are reassuring and indicate that higher levels of fitness are associated with a markedly attenuated incidence of coronary events regardless of the severity of coronary disease. Thus, coronary evaluation may inform prevention strategies in athletes that should include risk stratification, pharmacological, and lifestyle advice. In a majority of athletes, after excluding the presence of symptoms and inducible ischaemia, this advice should include encouragement to continue exercising.

## Declarations

### Data Availability

No data were generated or analyzed for or in support of this paper.
